# Rising trends in obesity and cancer-related mortality in the United States: a population-based analysis, 1999-2020

**DOI:** 10.1210/jendso/bvag006

**Published:** 2026-01-28

**Authors:** Faizan Ahmed, Tehmasp Rehman Mirza, Mohamed Bakr, Swapnil Patel, Mohammad Hossain

**Affiliations:** Department of Internal Medicine, Jersey Shore University Medical Center, Neptune, NJ 07753, USA; Department of Medicine, Shalamar Medical and Dental College, Lahore 54840, Pakistan; Department of Internal Medicine, Jersey Shore University Medical Center, Neptune, NJ 07753, USA; Department of Internal Medicine, Jersey Shore University Medical Center, Neptune, NJ 07753, USA; Department of Internal Medicine, Jersey Shore University Medical Center, Neptune, NJ 07753, USA

**Keywords:** obesity, cancer, mortality, CDC WONDER, epidemiology, public health

## Abstract

**Context:**

Obesity is a known risk factor for various cancers. Despite the growing prevalence of obesity in the United States, national-level trends in obesity-associated cancer mortality remain insufficiently quantified.

**Objective:**

To evaluate national trends in cancer mortality where obesity was listed as a contributing or underlying cause of death from 1999 to 2020.

**Methods:**

A retrospective cross-sectional study was conducted using publicly available mortality data from the CDC WONDER database. Adults aged 25 years and older were included if both obesity (ICD-10: E66) and cancer (ICD-10: C00–D9) were listed on their death certificates. Age-adjusted mortality rates (AAMRs) were calculated per 1 000 000 population, and Joinpoint regression was used to assess temporal trends through annual percent change (APC) and average annual percent change (AAPC).

**Results:**

A total of 33 572 obesity and cancer-related deaths were recorded. The overall AAMR rose from 3.73 in 1999 to 13.52 in 2020, with an AAPC of 5.92%. The highest mortality rates were observed in older adults, women, rural populations, and racial minorities, particularly Black and American Indian individuals. Breast, prostate, and lung cancers accounted for the greatest burden. A marked rise occurred between 2018 and 2020 across all subgroups.

**Conclusion:**

Obesity and cancer-related mortality in the United States has more than tripled over 2 decades, with notable disparities across demographic groups. These findings highlight the need for targeted public health efforts that integrate obesity prevention into cancer control strategies.

Obesity remains one of the most serious public health challenges in the United States, affecting over 40% of adults and contributing to the national burden of chronic disease and premature mortality. What was once primarily linked with cardiovascular and metabolic conditions, obesity is now firmly established as a major risk factor for both solid and hematologic malignancies, including breast, colorectal, endometrial, liver, prostate, thyroid, and hematopoietic cancers [1-4]. While cancer is increasingly outnumbering cardiovascular disease as the major cause of death in most high-income nations, growing evidence implicates obesity as a modifiable, upstream cause of rising cancer incidence and mortality [5, 6]. Obesity is identified by the Centers for Disease Control and Prevention (CDC) as a preventable cause of 13 forms of cancer, and it is estimated to be linked to 40% of cancer incidence in the United States [7].

Recent molecular-level research studies have identified the biological pathways through which obesity is associated with cancer formation. A pro-tumorigenic environment is regulated by insulin resistance, hormonal dysregulation, altered adipokine signaling, and chronic low-grade inflammation [4, 8]. Furthermore, recent studies demonstrate how immune dysfunction caused by obesity, such as impaired T-cell fitness and immune surveillance, can undermine the body's capability to recognize and destroy cancer cells [9, 10]. With consequences for the cancer prognosis and immunotherapy response, this immune dysregulation is now being recognized as a major contributor to obesity-related oncogenesis [10, 11]. Current population-level cancer prevention approaches do not fully account for the dynamic and multifaceted risk profile that spans the obesity-metabolic disease-cancer interface.

Although global level research and experimental literature on the underlying mechanisms are increasing, national-level surveillance data continue to be vital to understanding the complete magnitude of obesity's contribution to cancer death. In this CDC analysis, we examined US death certificate data from 1999 through 2020 to determine trends in obesity and cancer-related death in adults aged 25 years and older. Using ICD-10 codes and validated epidemiologic methods, we aimed to quantify how obesity has contributed to the cancer mortality burden over 2 decades. This study aims to inform future prevention policies, clinical risk assessments, and public health interventions by providing comprehensive, up-to-date evidence on a critical and preventable driver of cancer death in the United States.

## Methods

This study is exempted from institutional review board (IRB) approval because of the usage of a deidentified government-issued public use database.

### Data source

We obtained mortality data from the Centers for Disease Control and Prevention Wide-Ranging Online Data for Epidemiologic Research (CDC WONDER) Multiple Cause-of-Death Public Use database for the period January 1, 1999, through December 31, 2020 [12]. We identified death certificates that listed obesity and cancer as either underlying or contributing causes of death using the International Statistical Classification of Diseases, Tenth Revision (ICD-10 codes). We used codes E66 for obesity and C00-C97, and D00-D09 for cancer, which have been validated and widely employed in prior epidemiological literature to accurately classify both these conditions. The mortality data were collected for US adults aged ≥25 years at the time of death. Our reporting followed the Strengthening the Reporting of Observational Studies in Epidemiology (STROBE) guidelines [13].

### Data extraction

Information regarding deaths in patients with obesity and cancer, population sizes, years, demographics such as age, gender, and ethnicity or racial background, along with census regions, urbanization classifications, states, and place of death, was extracted. Race/ethnicity was coded according to the US Office of Management and Budget regulations. It was used as stated on death certificates, with the classifications being as non-Hispanic (NH) White, NH Black, NH American Indian or Alaska Native, NH Asian or Pacific Islander, and Hispanic or Latino [14]. Moreover, regions were defined per US Census Bureau classification as Northeast, Midwest, South, and the West [15]. The National Center for Health Statistics Urban-Rural Classification Scheme was used to categorize the populations. These statistics were based on the 2013 US Census classification. It comprises the urban population that includes large metropolitan areas with a population of ≥1 million, medium/small metro areas with a population of 50 000 to 999 999, and the rural regions with a population of <50 000 [16]. The place of death was categorized based on where the person died, such as in a hospital (inpatient, outpatient, emergency room), at home, in a hospice, nursing home, long-term care facility, or if they were dead on arrival or had an unknown location.

### Statistical analysis

To analyze national trends in patients with concomitant obesity and cancer, we calculated crude mortality rates (CMRs) and age-adjusted mortality rates (AAMRs) per 1 000 000 population from 1999 to 2020. Rates were stratified by age groups, year, gender, race/ethnicity, census regions, states, urban-rural classification, and place of death. We calculated the rates using 95% CIs to show the range in which the true values are likely to fall. To find the CMRs, we divided the number of deaths caused by both obesity and cancer by the total US population for that year. For AAMRs, we adjusted the death counts based on the age distribution of the US population in the year 2000 [17]. To analyze how these rates changed over time, we used the Joinpoint Regression Program (version 5.2.0) from the National Cancer Institute [18]. This software helped us calculate the annual percent change (APC) in AAMRs and the 95% CIs for these changes. The program uses log-linear regression models to identify when significant shifts occurred in the trend. We used a statistical method called the Weighted BIC test to find the APCs for each time segment between these change points (joinpoints). To decide if the changes in mortality were increasing or decreasing, we applied a 2-tailed *t* test. A *P* value of .05 or less was considered statistically significant, meaning the change was unlikely to have occurred by chance.

## Results

### Overall obesity and cancer

**Figure 1:**
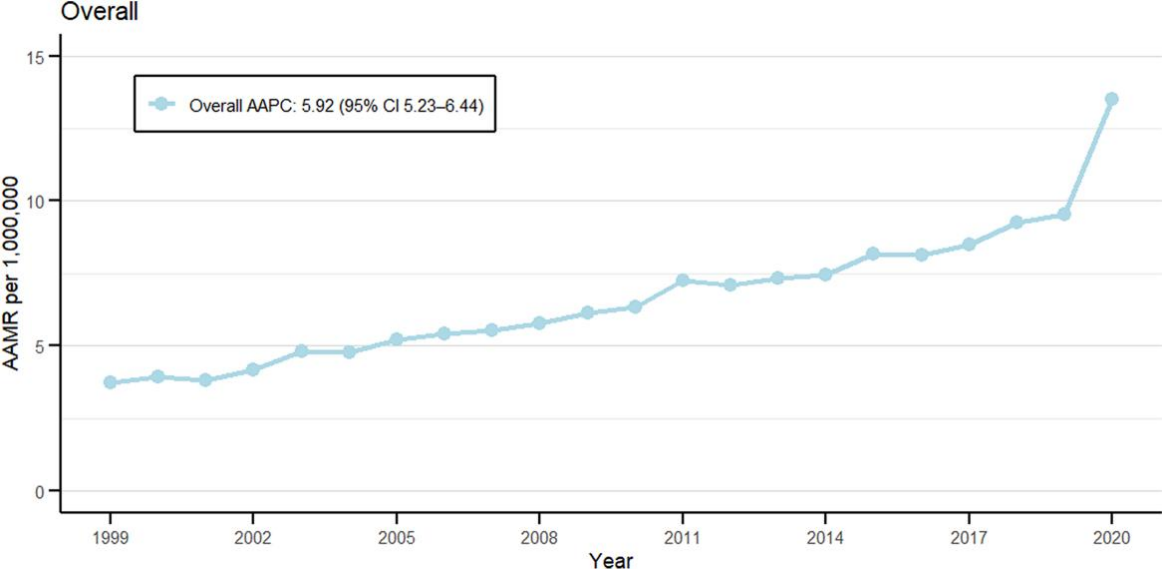
Overall obesity and cancer-related AAMR per 1 000 000 in the United States, 1999 to 2020.

Obesity and cancer were associated with a total of 33 572 deaths in the United States from 1999 to 2020 (Table 1). The age-adjusted mortality rates (AAMR) per million ranged between 3.73 (95% CI: 3.45-4.01) in 1999 to 13.52 (95% CI: 13.08-13.97) in 2020. The highest AAMR per million was reported in 2020 at 13.52 (Fig 1, Table S2 [19]). The average AAMR was recorded to be 6.91 per million (95% CI: 6.84-6.99) for years 1999-2020. The rates demonstrated major variations in these years with the average annual percentage change (AAPC) of 5.92 (95% CI: 5.23-6.44, *P* < .000001). The major upward spike was observed from 2018 to 2020 with the annual percentage change (APC) of 19.37 (95% CI: 9.59-24.19, *P* < .000001) (Table S3 [19]).

**Table 1:** Baseline characteristics of obesity and cancer-related mortality trends in the United States from 1999 to 2020

	Total deaths	Age-adjusted mortality rate		Average annual percentage change	
	N (%)	1999	2020	1999-2020	*P*
Overall	33 572	3.73	13.52	5.92	<.01
**Sex**					
Male	14 585 (43.4)	3.44	13.38	6.75	<.01
Female	18 987 (56.6)	3.98	13.74	5.37	<.01
**Race/Ethnicity**					
NH Black	4465 (13.3)	5.12	18.94	5.37	<.01
NH White	26 525 (79.0)	3.80	14.06	6.18	<.01
Hispanic	1986 (5.9)	2.04	9.75	6.31	<.01
**Census region**					
Northeast	5288 (15.8)	3.63	11.63	5.56	<.01
Midwest	8561 (25.5)	4.21	15.53	6.01	<.01
South	11 565 (34.4)	3.31	13.40	6.59	<.01
West	8158 (24.3)	3.95	13.39	5.41	<.01
**Age groups**					
<65	14 305 (42.6)	1.90	6.71	5.44	<.01
>65	19 267 (57.4)	11.26	41.54	6.26	<.01
**Urbanization**					
Metro	25 732 (76.6)	3.62	12.73	6.03	<.01
Non-metro	7840 (23.4)	4.07	17.99	6.98	<.01

Abbreviation: NH, non-Hispanic.

### Gender

**Figure 2:**
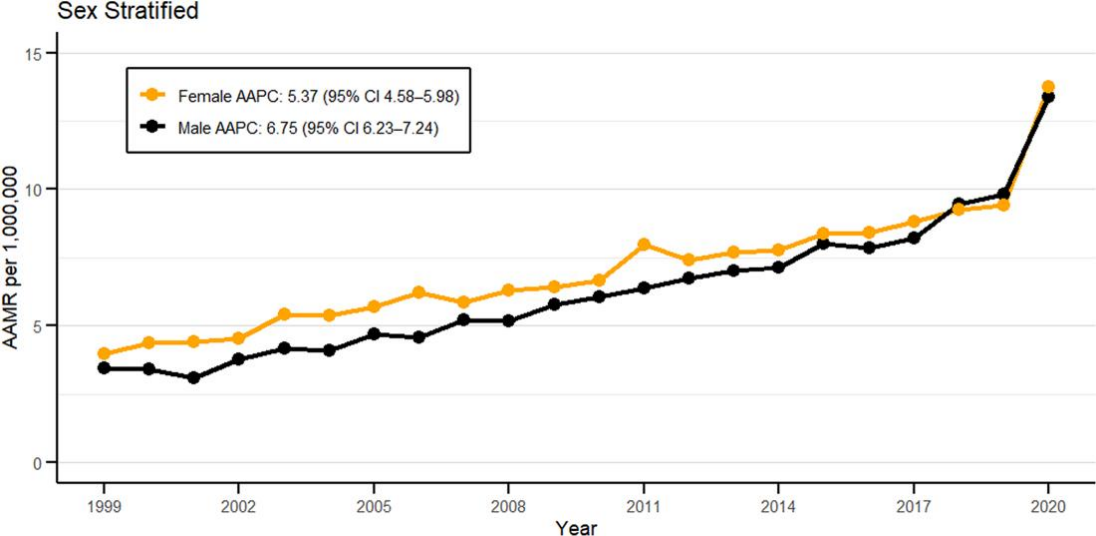
Sex-stratified obesity and cancer-related AAMRs per 1 000 000 in the United States, 1999 to 2020.

Segregation of the database by gender revealed similar statistics for both groups but the female population had higher AAMR compared to male population. A total of 18 987 deaths occurred in females and 14 585 deaths in males from 1999 to 2020. (Figure 2, Table S1 [19]). The average AAMRs for female individuals was recorded to be 7.22 per million (95% CI: 7.12-7.32) and 6.59 per million (95% CI: 6.48-6.7) for male individuals. The highest AAMR per million for the female and the male groups was recorded in 2020 at 13.74 (95% CI: 13.12-14.35) and 13.38 (95% CI: 12.73-14.04) respectively (Table S2 [19]). Both female and male populations had an increase in mortality trends from 1999 to 2020 with AAPC of 5.37 (95% CI: 4.58-5.98, *P* < .000001) and 6.75 (95% CI: 6.23-7.24, *P* value <.000001), respectively, with the steepest mortality trends observed from 2018-2020 at APC 19.16 (95% CI: 7.96-25.34, *P* < .000001) and 19.86 (95% CI: 11.87-24.10, *P* < .000001) (Table S3 [19]).

### Age groups

**Figure 3:**
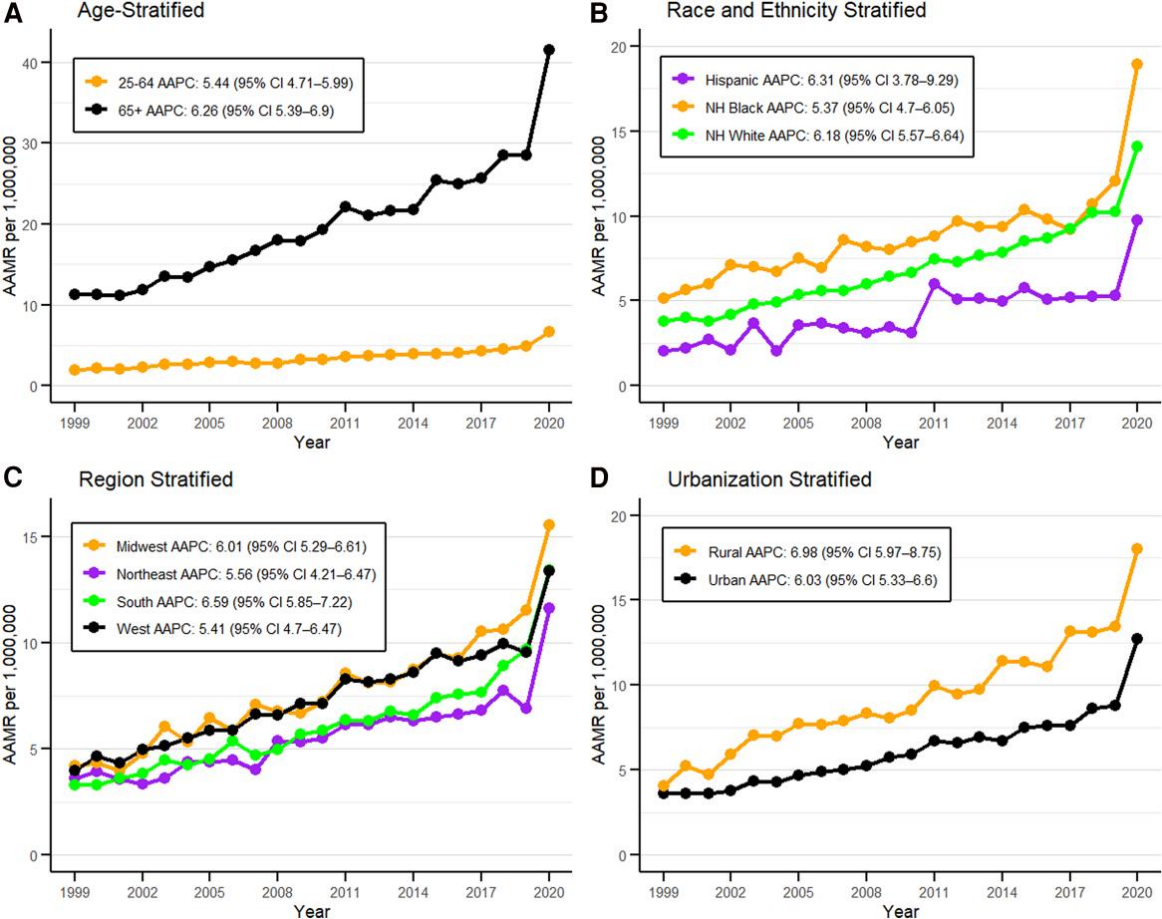
obesity and cancer-related AAMR per 1 000 000 deaths in the United States, 1999 to 2020, stratified by (A) age, (B) race/ethnicity, (C) census region, and (D) urbanization.

The mortality trends for obesity and cancers were divided into 2 age groups, the younger population (25-64 years), and the older population (65+ years). A total of 14 305 deaths were reported in the younger population between 1999 and 2020. The average AAMR was 3.54 per million (95% CI: 3.48-3.59), with the highest rate recorded in 2020 at 6.71 per million (95% CI: 6.35-7.08). In the older population, there were 19 267 deaths during the same period. The average AAMR was 20.82 per million (95% CI: 20.52-21.11), and the highest AAMR was observed in 2020 at 41.54 per million (95% CI: 39.82-43.26) (Fig 3A, Table S4 [19]). These trends showed that the highest mortality rates were observed in the age groups above 65 years in 1999-2020 with the AAPC 6.26 (95% CI: 5.39-6.90, *P* < .000001) (Table S2 [19]).

### Race/ethnicity

Obesity and cancer-related mortality trends were segregated by ethnicity into 2 main categories: *Hispanic or Latino* and *non-Hispanic origin* which include 4 races (American Indians or Alaska Native, Asian or Pacific Islander, Black or African American, White). Among the non-Hispanic (NH) population, from 1999-2020 the average AAMR per million for NH American Indian was reported as 9.04 (95% CI: 7.9-10.18) with the highest being in 2019 recorded at 16.9 (95% CI: 11.14-24.59); for NH Asian the average AAMR per million was 1.26 (95% CI: 1.1-1.41) with the highest being in 2020 reported at 2.05 (95% CI: 1.38-2.92); for NH Black 9.2 (95% CI: 8.92-9.47) with the highest being in 2020 recorded at 18.94 (95% CI: 17.32-20.56); for NH White 7.13 (95% CI: 7.04-7.22) with the highest being in 2020 recorded at 14.06 (95% CI: 13.52-14.61); and with the overall average AAMR for the NH population was 7.11 per million (95% CI: 7.03-7.18) for 1999-2020. For Hispanic or Latino, the average AAMR per million was recorded at 4.66 (95% CI: 4.45-4.87) in years 1999-2020 with the highest being reported in 2020 at 9.75 (95% CI: 8.58-10.92) (Fig 3B, Table S5 [19]). All the races (American Indian, Asian, Black, White, Hispanic) showed a rise in age-adjusted mortality trends from 1999-2020, with the Hispanic group having the highest AAPC of 6.31 (95% CI: 3.78-9.29, *P* < .000001) and the Black group having the least AAPC of 5.37 (95% CI: 4.70-6.05, *P* < .000001) (Table S2 [19]).

### Cancer type stratification

**Figure 4:**
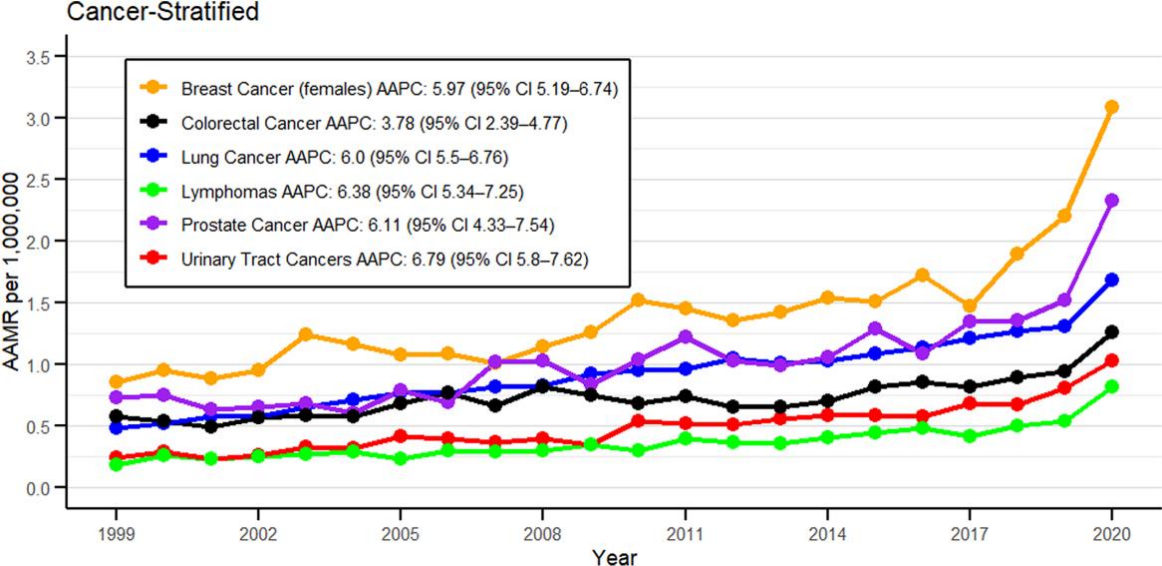
Obesity and cancer-related AAMR per 1 000 000 stratified by cancer types in the United States, 1999 to 2020.

**Figure 5:**
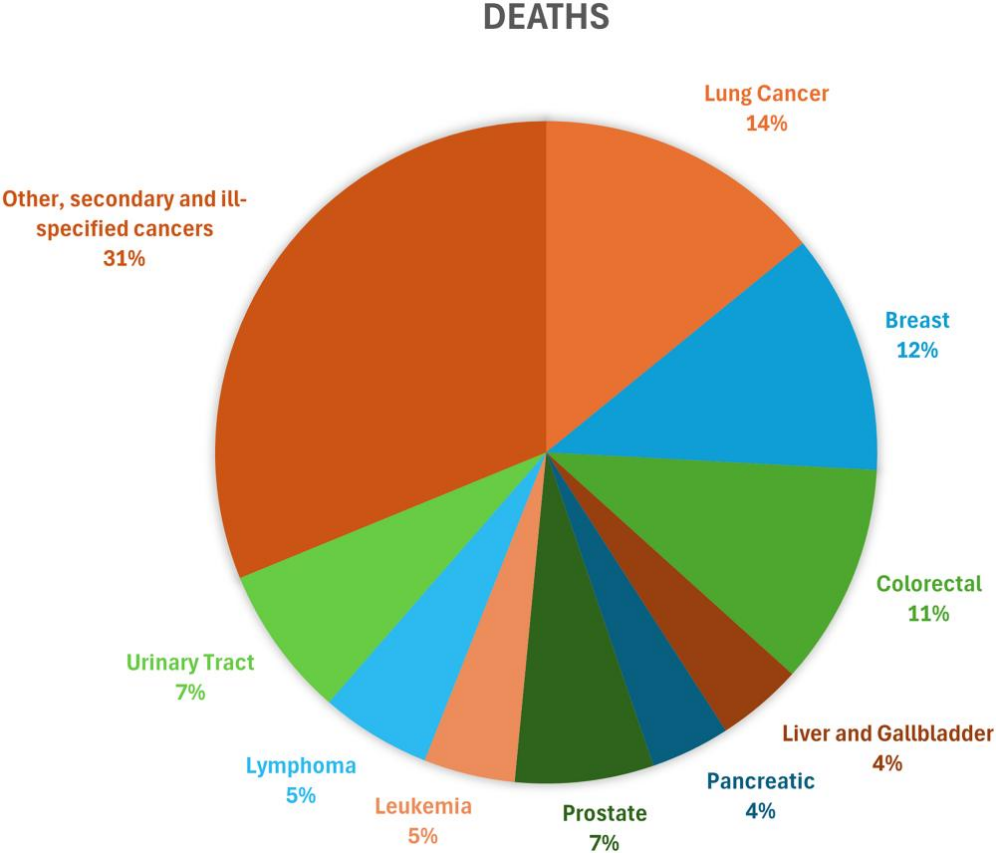
Proportion of deaths attributed to each cancer type in adults with obesity in the United States, 1999 to 2020.

Mortality trends were further explored on a cancer type basis; there was a clear site-specific variation seen in the burden from obesity and cancer-related deaths. Of all malignancies, breast cancer in females had the highest average AAMR of 1.40 per million (95% CI: 1.30 to 1.50) reaching a peak of 3.09 per million in 2020 and a 12% proportion in total deaths (Fig 4 and 5, Table S6 [19]). Lung cancer proved to have had a persistent burden, showing an average AAMR of 0.92 per million (CI: 0.79 to 1.04), and a peak mortality rate of 1.68 per million in 2020 with a 14% proportion in absolute deaths. Colorectal cancer had an average AAMR of 0.73 per million (95% CI: 0.66-0.81) with the highest rate of 1.01 per million observed in 2020. Among all of them, a sharp increase was noted in 2020. Breast cancer showed the highest rise from 2018 to 2020 with an APC of 32.37 (95% CI: 17.34-41.40, *P* < .000001), while urinary tract cancer showed the highest rise throughout the study period with an AAPC of 6.79 (95% CI: 5.80-7.62, *P* < .000001) (Fig. 4, Table S2 [19]).

### Geographical regions (census & states)

The highest number of casualties were reported in the Southern census region (11 565 deaths) followed by the Midwest (8561 deaths), West (8158 deaths), and Northeast (5288 deaths) from 1999 to 2020 (Table S1 [19]). The highest average AAMR per million was recorded in the Midwest at 7.96 (95% CI: 7.79-8.13) while the Northeast region had the lowest, recorded at 5.7 (95% CI: 5.54-5.85) in 1999-2020 (Fig 3C, Table S7 [19]). All the regions had a rise in age-adjusted mortality trends from 1999-2020: the Northeast (AAPC 5.56 [95% CI: 4.21-6.47], *P* < .000001); Midwest (AAPC 6.01 [95% CI: 5.29-6.61], *P* < .000001); South (AAPC 6.59 [95% CI: 5.85-7.22], *P* < .000001); and West (AAPC 5.41 [95% CI: 4.7-6.47], *P* < .000001). Notably, the West showed significant decline in mortality trends from 2015-2018 at APC −0.32 (95% CI: −4.38 to 4.77, *P* = .92) followed by significant incline from 2018-2020 (Table S2 [19]).

**Figure 6:**
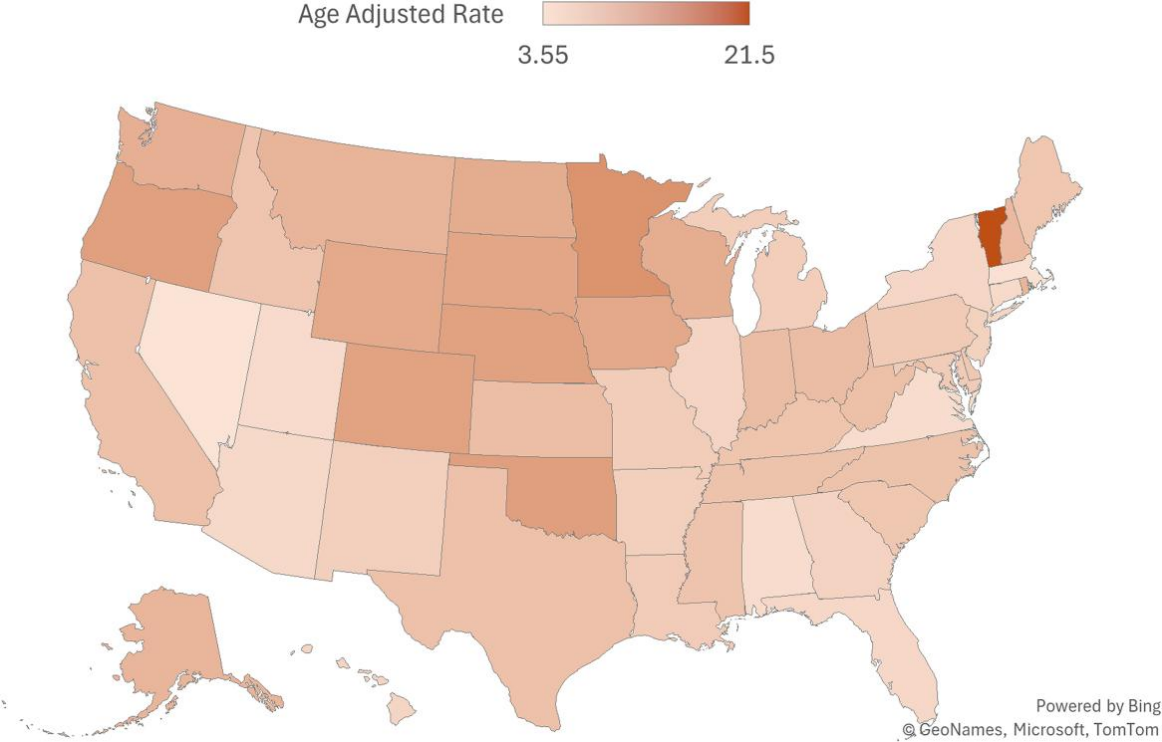
State-stratified obesity and cancer-related AAMRs per 1 000 000 in United States 1999-2020.

From 1999-2020, the highest number of deaths related to obesity and cancers were reported in California, which was 4061 deaths with AAMR at 7.62 per million (95% CI: 7.39-7.86) and the lowest (49 deaths) in District of Columbia with the AAMR at 5.54 per million (95% CI: 4.09-7.35) (Fig 6, Table S8 [19]). The states having mortality rates at or above 90th percentile are Vermont, Minnesota, Oklahoma, Oregon, and Nebraska. The states having mortality rates at or below 10th percentile are Utah, Alabama, Virginia, Massachusetts, Nevada (Table S9 [19]).

### Urbanization

Segregation of the database by urbanization revealed that in the years 1999-2020 urban areas had the larger number of deaths (25 732) with the average AAMR 6.4 per million (95% CI: 6.32-6.48) and the highest AAMR reported in 2020 at 12.73 per million (95% CI: 12.26-13.2). Similarly, rural areas had 7840 deaths in these years with the average AAMR of 9.45 per million (95% CI: 9.24-9.66) and the highest AAMR reported in 2020 at 17.99 per million (95% CI: 16.67-19.32) (Fig 3D, Table S10 [19]). Both urban and rural areas had an overall increase in mortality trends from 1999-2020 with AAPCs of 6.03 (95% CI: 5.33-6.60, *P* < .000001) and 6.98 (95% CI: 5.97-8.75, *P* < .000001), respectively. Rural areas showed no significant change in mortality trends from 2003-2018 with an APC of 4.40 (95% CI: −3.86 to 5.14, *P* = .24) followed by a significant incline from 2018-2020 with an APC of 16.41 (95% CI: 5.97-23.66, *P* < .000001) (Table S2 [19]).

### Place of death

**Figure 7:**
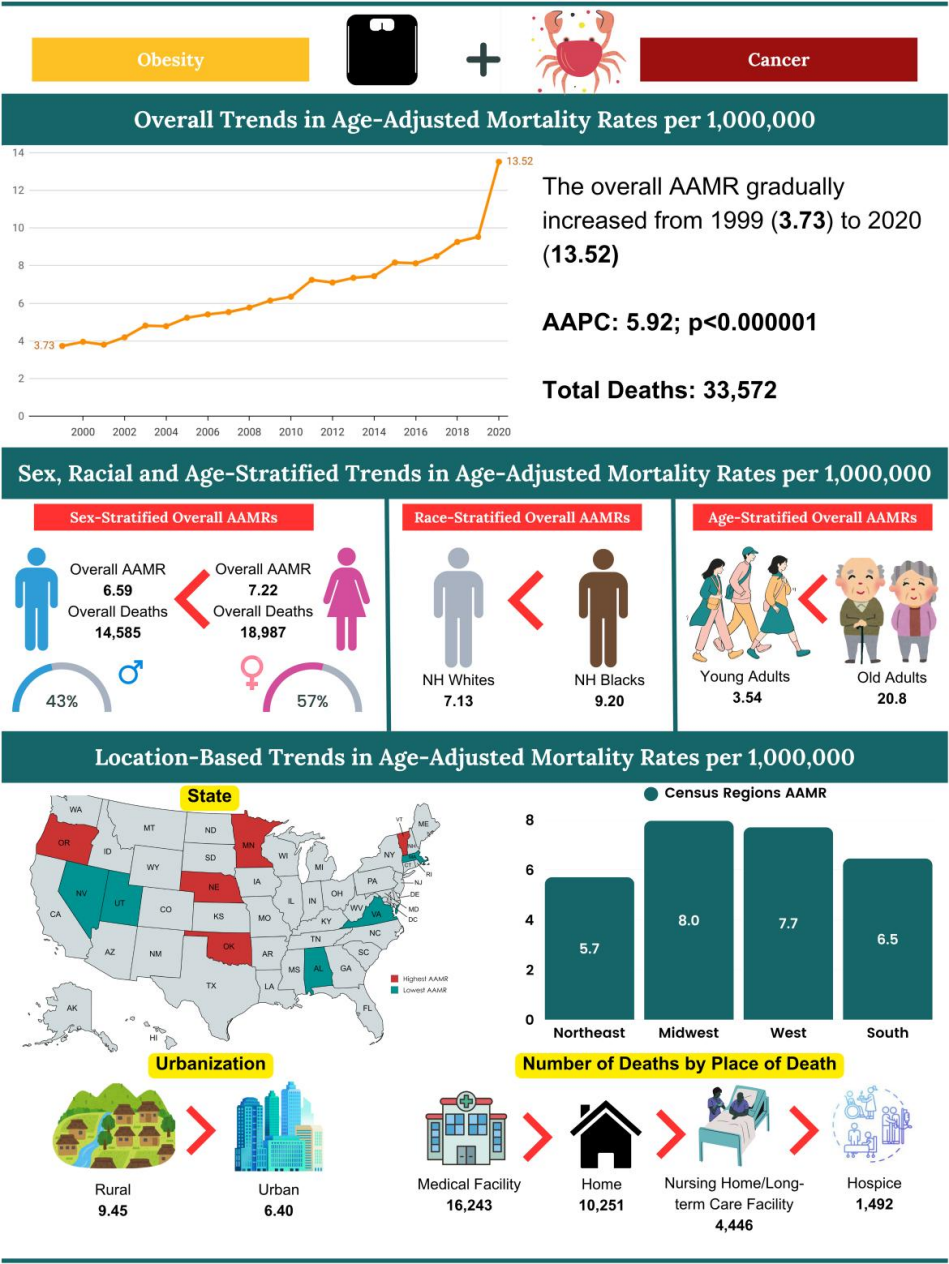
Central illustration: demographic profiles in obesity and cancer-related mortality among adults 25 to 85+ in the United States, 1999 to 2020.

Database trends from 1999-2020 showed that Medical Facility-Inpatient had the greatest number of deaths (13 373) and proportion of total deaths (39.8%), followed by decedent's home (10 251, 30.5%), Nursing Home/Long term care (4446, 13.3%), Medical Facility–Outpatient or ER (2636, 7.9%), Hospice Facility (1492, 4.4%), Others (1084, 3.2%), Medical Facility–Dead on Arrival (217), Place of death unknown (56), Medical Facility–Status Unknown (17) (Fig 7, Table S11 [19]).

## Discussion

This study underscores critical and alarming insights into the persistent and growing pattern of obesity and cancer-related mortality among the adult US population. According to the American Cancer Society, overall cancer mortality has greatly declined [20] but obesity and cancer-related mortality has shown a sustained and disproportionate rise [11] as values tripled from the initial figures, which highlights the complex interplay between obesity prevalence and its carcinogenic consequences [21, 22], against the backdrop of evolving screening, treatment, and preventive efforts.

Obesity is a recognized risk factor for almost 13 types of cancers [21] as it promotes chronic low-grade inflammation, hyperinsulinemia, increased estrogen and androgen production from adipose tissue, and altered immune surveillance—all of which contribute to tumor initiation, progression, metastasis [23], and poor prognosis of breast, colorectal, and pancreatic cancers ​ [24], as is reflected by the results of this study.

Gender-specific mortality trends exhibited variations; the combined mortality burden was greater in women as compared to men. This could reflect the overwhelming contribution of breast cancer, which is both high-burden and highly obesity-sensitive. It also raises intriguing hypotheses around gender-specific interaction between adiposity, hormones, tumor biology, access to cancer screening, and health-seeking behavior, which deserves further exploration [25-27].

Racial and ethnic disparities were also evident. The highest burden of obesity and cancer-related mortality was seen in NH Black individuals—an alarming trend that likely stems from intersecting factors such as structural racism, delayed diagnosis, and inequitable access to high-quality cancer care [28]. Hispanic and Native American populations also showed concerning increases, potentially linked to rapid shifts in lifestyle patterns, urbanization, and socioeconomic challenges [29].

Regional disparities were also seen as the greatest mortality burden borne by the Southern states and California. Urbanization trends also illustrate different cancer outcomes. The mortality patterns were significantly variable among the rural and urban population; rural counties reported higher and rising AAMRs which may be due to reduced access to preventive healthcare infrastructure as well as higher rates of risk factors such as obesity and smoking [30].

Importantly, the treatment-related factors also play an important role toward the high mortality as they further exacerbate outcome disparities. The response to certain chemotherapy agents is different among patients with obesity; for example, in breast cancer, studies have shown reduced pathological complete response to neoadjuvant anthracycline-taxane-based regimens in women with obesity [31]. Similarly, pharmacokinetic concerns have been raised for drugs such as docetaxel, with altered drug distribution and increased toxicity risk when dosed per body surface area without appropriate obesity-specific adjustments [32]. These treatment dynamics may contribute not just to lower efficacy but also to higher rates of dose reduction, delays, or early discontinuation.

Complicating matters further is the prevalence of comorbidity in populations with obesity, such as type 2 diabetes [1], cardiovascular disease, and metabolic syndrome, which can limit surgical eligibility or tolerance for chemotherapy/radiotherapy and significantly increase the risk of postoperative or treatment-related complications. In women with breast cancer, comorbid obesity and diabetes have been independently associated with higher cancer-specific mortality [1].

Our analysis pointed toward the notable variation in the obesity and cancer-related mortality across subtypes. Breast cancer was found to be more prevalent than other cancer types in contributing to obesity and cancer-related mortality, which aligns with existing evidence as individuals with obesity, especially women, are documented to have lower mammography screening rates, due in part to stigma, logistical imaging limitations, and avoidance of healthcare settings [33]. This results in diagnosis at more advanced stages, reduced survival, and increased treatment complexity. Furthermore, breast imaging is technically challenging, with lower sensitivity in mammograms, leading to missed or delayed diagnoses [34]. Although lung cancer is traditionally less associated with obesity as compared to smoking, this study highlighted obesity as a potential cofactor in lung cancer mortality particularly through inflammatory and immuno-metabolic pathways as previous studies illustrated [35]. Urinary tract cancers and leukemia mortality trends also showed upward trajectory pointing to a significant association [36, 37].

Most of the obesity and cancer-related deaths occurred in patient settings suggesting associated complications and late-stage presentations.

The fact that breast cancer mortality peaked in 2020 also reflects compounded effects of delayed screening during the COVID-19 pandemic, underscoring the importance of maintaining preventive care continuity during health crises.

Our study benefits from the use of nationally representative mortality data for 2 decades allowing robust data analysis and cancer type stratification and suggests the urgent need to integrate obesity prevention strategies into national cancer control programs. Tailored, cancer-specific public health policies, such as targeted screening, lifestyle interventions, and early detection efforts, are also crucial to reduce the obesity and cancer-related mortality across specific groups.

### Limitations

Analysis was done based on observational mortality data which was obtained from death certificates. It, therefore, cannot determine a causal relationship between obesity and cancer-related mortality. The existence of obesity and cancer in a death certificate does not always mean a direct causal relationship. Misclassification and underreporting in death certificate records are possible, especially in cases of obesity, which are inconsistently documented as a contributing cause of death. This may result in an underestimation of cancer deaths that were associated with obesity. Using ICD-10 code E66 could be insufficient in portraying the entire spectrum of obesity-related risk. It captures only those instances where obesity was documented explicitly, which may have excluded overweight individuals or those having metabolic dysfunction who were not characterized as being obese. Detailed clinical and lifestyle information such as the body mass index, whether a patient smokes or not, the degree of physical activity, availability of treatment, socioeconomic status, and comorbidities are not available in the publicly available database, although this information can substantially influence cancer mortality outcomes independently. This analysis views obesity as a binary condition, without accounting for changes in body mass index over time or variations seen in degrees of obesity, such as Class I vs Class III obesity. Population-level data was used in the subgroup analyses that might not have reflected risk association at the individual level. Use of aggregated data can be misleading when analyzing individuals. The trends cover a period of more than 20 years, and during that period, both the diagnostic process and documentation of obesity may have changed. Our study may have introduced reporting biases due to regional differences in access and documentation, along with standards of healthcare across states and urban-rural gradients.

## Conclusion

This nationwide population-based analysis of US mortality data from 1999-2020 established that there was a sharp increase in mortality where obesity and cancer were listed as contributing causes. The overall age-adjusted mortality rate (AAMR) rose to 13.52/million from 3.73 with an average annual percentage change (AAPC) of 5.92 (95% CI: 5.23-6.44, *P* < .000001). This increase was especially marked in recent years, between 2018 and 2020 (APC: 19.37%, 95% CI: 9.59-24.19). The results of subgroup analyses showed that mortality was higher in females, Black and American Indian populations, people aged 65 years or older, and those who were living in rural areas and in the Southern and Midwestern states of the United States. These trends reflect the unequal burden of obesity and cancer-associated mortality among socioeconomically and geographically marginalized groups.

Such findings emphasize the dire need to work on public health strategies that integrate cancer control with obesity prevention. Interventions should focus on populations at higher risk and remove structural barriers to health equity, especially in underserved and rural areas. Future research is warranted to investigate causal factors, site-specific associations, and to study the effect of weight loss interventions on long-term cancer survival.

## Data Availability

Original data generated and analyzed during this study are included in this published article or in the data repositories listed in References.

## Abbreviations

AAMR: age-adjusted mortality rate
AAPC: average annual percentage change
APC: annual percent change
CDC: Centers for Disease Control and Prevention
CMR: crude mortality rate
ICD: International Statistical Classification of Diseases
NH: non-Hispanic
